# Impact of COVID-19 on the oral health of adults in Wuhan and China: results of a nationwide online cross-sectional questionnaire survey

**DOI:** 10.1186/s12903-021-01533-z

**Published:** 2021-03-26

**Authors:** Shuang Zhang, Chang Liu, Chenzheng Zhang, Han Jiang, Baojun Tai, Minquan Du

**Affiliations:** The State Key Laboratory Breeding Base of Basic Science of Stomatology (Hubei-MOST) and Key Laboratory of Oral Biomedicine Ministry of Education, School and Hospital of Stomatology, Wuhan University, Wuhan, 430079 China

**Keywords:** Oral health, COVID-19, Questionnaire survey

## Abstract

**Background:**

COVID-19 has seriously threatened the health and lives of people. This study aimed to investigate the impact of COVID-19 on the oral health of adults in Wuhan and other places of China amid the epidemic and to evaluate attitudes towards dental care in the post-epidemic period.

**Methods:**

An online cross-sectional survey based on a questionnaire of 22 questions was conducted.

**Results:**

A total of 3352 valid questionnaires were collected. Participants from Wuhan tended to be relatively psychologically affected and more concerned about future dental treatment. Toothbrushing frequency did not differ significantly between participants from Wuhan and other places and was associated with the prevalence of oral problems people encountered. Gingival bleeding, bad breath and oral ulcers were the three most common oral problems amid the epidemic, and significantly more participants in Wuhan experienced oral problems than other places in China.

**Conclusion:**

The three most common oral problems amid the epidemic were gingival bleeding, bad breath and oral ulcers. Adults in Wuhan tended to be more seriously affected and suffered more oral problems than people from other places in China. Maintaining good oral health behaviours plays an important role in preventing dental problems. It is crucial to establish and to follow the standard guidelines for the provision of dental care during and after the epidemic.

**Supplementary Information:**

The online version contains supplementary material available at 10.1186/s12903-021-01533-z.

## Background

In December 2019, a cluster of cases of pneumonia with unknown aetiology were reported in Wuhan, the capital city of Hubei Province, China [1]. This disease has spread rapidly across the country and all over the world since then. A novel coronavirus was eventually identified, and the WHO declared the novel coronavirus outbreak a Public Health Emergency of International Concern (PHEIC) on 30 January 2020 [2]. This is the 6th time that the WHO has declared a PHEIC since the International Health Regulations came into force in 2005. In February 2020, this virus was named severe acute respiratory syndrome coronavirus-2 (SARS-CoV-2), and the epidemic disease was announced as coronavirus disease 2019 (COVID-19) [3, 4]. As the epidemic continued to spread worldwide with severe consequences, the WHO officially characterized COVID-19 as a pandemic on 11 March 2020 [5]. According to the latest WHO COVID-19 situation report (15 December 2020), a total of 71,351,695 confirmed cases were reported worldwide, and 1,612,372 people have lost their lives [6]. There is no specific medication for this virus so far, and although the development of vaccines is already in progress, there is still a long road to large-scale application [7]. Together, these factors have brought deep public concern.

The whole city of Wuhan, the earliest epicentre of this epidemic, unprecedentedly announced a “lockdown” on 23 January 2020, two days after the confirmation that this new disease could be transmitted from human to human. The government took a series of strong measures to stop the transmission of the virus to the greatest extent. People could not leave Wuhan, were asked to stay at home as much as possible and to follow the strict rules of social distancing. Soon, similar measures were implemented in other provinces of the nation as the epidemic began to spread across the country. Although the 76-day-long lockdown of the city ended on April 8, citizens were still advised to minimize outings and public gatherings. People’s daily lives have not yet returned to normal.

COVID-19 and the subsequent unprecedented lockdown measures have greatly affected physical and mental status [8, 9], and daily behaviours, including oral healthcare behaviours, may have changed as a result. Moreover, regular medical activities were critically disrupted amid the pandemic. It is particularly noteworthy that most dental institutions in China suspended general dental services and are providing emergency dental services only, as dental procedures generate many droplets and aerosols leading to the transmission of pathogens [10, 11]. Therefore, we are concerned about the oral health of the public amidst the epidemic. It is very important to explore the impact of COVID-19 on oral health, i.e., oral healthcare behaviours and status, the dental problems encountered, how people dealt with these problems, and whether these problems aggravated their uneasiness and anxiety.

Thus, in this study, we conducted an online questionnaire-based cross-sectional survey to investigate the impact of COVID-19 on the oral health of adults in Wuhan and other places of China amid the epidemic and to evaluate attitudes towards dental care in the upcoming post-epidemic period.

## Methods

### Study design and population

This survey was conducted through the most popular instant messaging software “WeChat” or Web link (https://www.wjx.cn/jq/73967242.aspx) and sent to adults in Wuhan or other places of China.

The survey period was from 24 January 2020 to 2 May 2020, and a total of 3352 adult participants completed the questionnaire. Participants were informed that participation was anonymous and voluntary.

### Measures

The questionnaire used in this survey was developed for this study (Additional file 1) and proposed based on the results of a pilot study, consisting of questions regarding the following aspects: general information (e.g., gender and age), psychological status, living habits, oral health behaviour, oral health status, and knowledge and attitudes towards nosocomial infections and future dental treatment.

Specifically, general information included gender, age and residing location during the epidemic. Participant psychological status amid the epidemic was evaluated by four questions referring to the commonly used Self-Rating Anxiety Scale (SAS). The four questions are worded about increasing anxiety levels, and each question is scored on a scale of 1–4 (none or a little of the time, some of the time, a good part of the time, and most of the time). Total scores were calculated to evaluate psychological status. Living habits included daily work and rest time, dining frequency, smoking and drinking habits. Toothbrushing frequency was assessed as information about each participant’s oral hygiene behaviour. Participants were also asked about the dental problems they encountered, the way they dealt with the problems and the concern of not receiving timely dental care. The last three questions concentrated on knowledge and attitudes towards nosocomial infections and future dental treatment.

### Statistical analysis

Data were collected and statistically analysed with SPSS 22.0 (SPSS, Chicago, IL). Comparisons were performed using Student’s t-test and the chi-square test. A *p*-value < 0.05 was required for significance.

## Results

We obtained a total of 3352 valid questionnaires in this survey. A total of 1217 (36.31%) participants were male, and the rest were female. Regarding age, 1241 (37.02%) participants were between 18 and 30 years old, and 1582 (47.19%) were between 31 and 50 years old. The rest (529, 15.79%) of the study participants were over 51 years old. Nearly half (1507, 44.96%) of the participants stayed in Wuhan amidst the epidemic.

The scores indicating psychological status were 6.94 ± 2.225 for participants in Wuhan and 6.18 ± 1.889 for participants in other places in China, and this difference was significant.

### Impact of Covid-19 on oral health behaviour

A total of 58.29% of the study population changed their work and rest times during the epidemic. Compared with other places in China, more participants in Wuhan changed their work and rest times, with rates of 62.84% and 54.57%, respectively. Regarding dining frequency, 33.38% of the participants changed their dining frequency, with 8.92% experiencing more meals and 24.46% experiencing fewer meals. Similar to the trends in work and rest time changes, more people (40.30%) in Wuhan changed their dining frequency than in other places of China, with 8.95% and 31.39% experiencing greater and lesser numbers of dining times, respectively.

A total of 427 of the study population were smokers. A total of 28.33% of these individuals smoked more than usual, 17.80% smoked less, and the rest remained the same. Notably, 36.3% of the smokers in Wuhan smoked more cigarettes than they usually did, which was higher than the nationwide level. A total of 611 of the participants had a habit of drinking, and 27.33% of them drank more amidst the epidemic while 28.31% drank less. Specifically, 34.59% of participants in Wuhan drank more and 25.18% less.

Table 1 shows the age-specific toothbrushing frequency of participants in Wuhan and other places in China. A total of 73.09% of the participants brushed their teeth twice or more a day, 23.82% brushed their teeth once, and the rest did not brush teeth every day. Toothbrushing frequency did not differ significantly by age among participants in Wuhan and other places.

**Table 1:** Toothbrushing frequency of participants in Wuhan and other places of China

	Population	Toothbrushing frequency (%)		
		Twice or more	Once	Less than once
Wuhan	1507	73.59	23.62	2.79
Other places	1845	72.68	24.82	2.50
Total	3352	73.09	24.28	2.63

### Impact of Covid-19 on oral disease

**Fig. 1:**
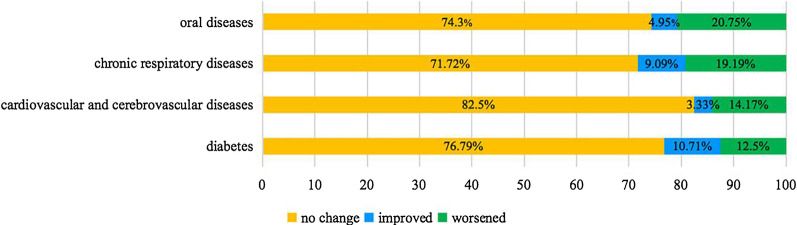
Self-assessment of the changes in the original diseases amid the epidemic

Participants were asked about their history of diabetes, cardiovascular and cerebrovascular diseases, chronic respiratory diseases and oral diseases before the epidemic and made self-assessments of the changes in the original diseases during the epidemic (worse, better, or no change). The results showed that 20.75% of participants with a history of oral disease thought that the disease worsened, which was the highest proportion compared with that of other diseases (Fig. 1).

**Fig. 2:**
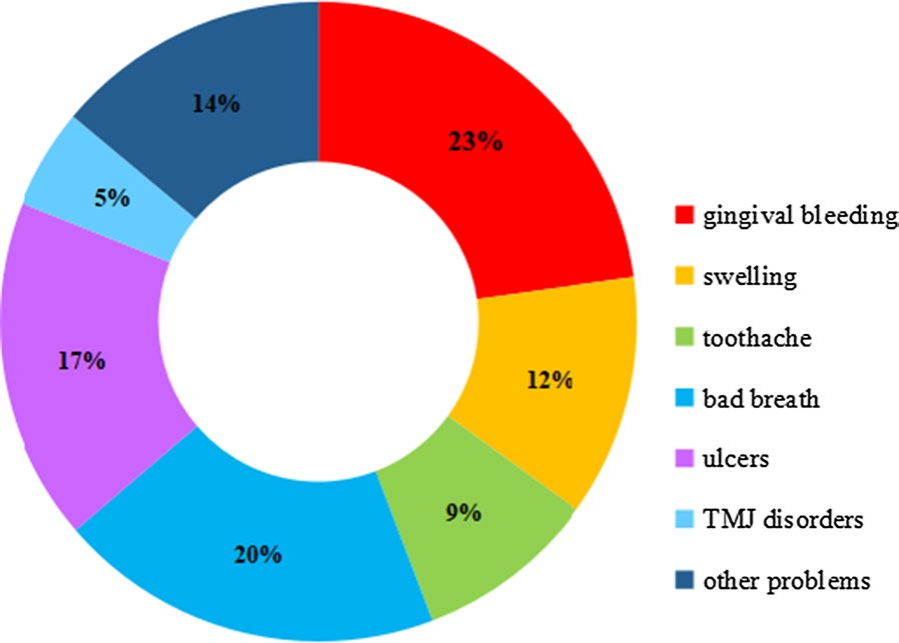
Oral problems amid the epidemic

A total of 59.87% of the participants had oral problems during the epidemic. Significantly more participants in Wuhan (63.30%) experienced oral problems than other places in China (57.07%). Figure 2 shows the specific problems the total population experienced. The three most common oral problems amidst the epidemic were gingival bleeding (23%), bad breath (20%) and oral ulcers (17%).

The relationship between toothbrushing frequency and oral problems is shown in Table 2. The prevalence of five common oral problems tended to decrease as the participants brushed their teeth more.

**Table 2:** Toothbrushing frequency and prevalence of oral problems

	Population	Prevalence of certain oral problems (%)					
		Gingival bleeding	Bad breath	Ulcers	Swelling	Toothache	TMJ disorder
Twice or more	2450	22.08*	17.18*^#^	16.37*	11.76*^#^	8.20*^#^	5.14
Once	814	22.60*	23.83*	18.92*	12.78	11.18	4.42
Less than once	88	37.50	38.64	25.00	17.05	15.91	6.82

*p < 0.05 compared with the “less than one” group; ^#^*p* < 0.05 compared with the “once” group

**Fig. 3:**
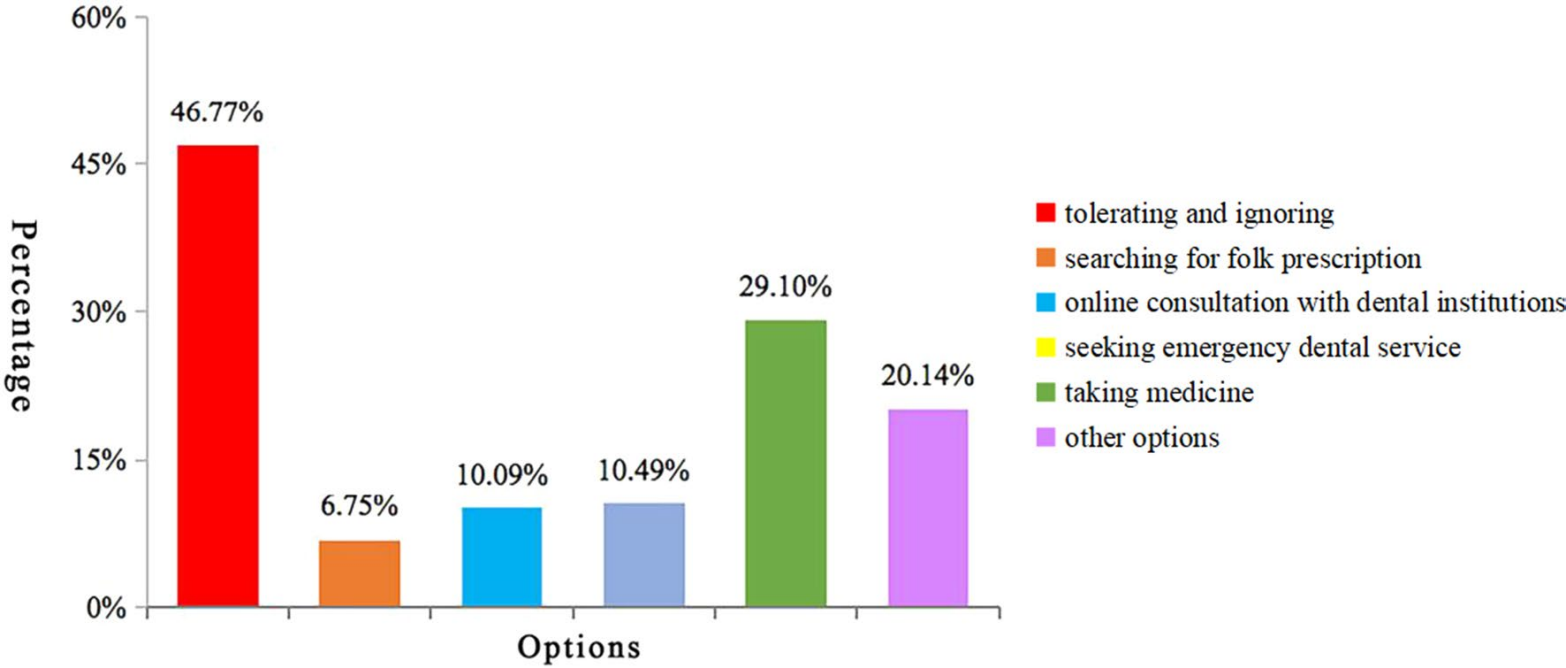
Different options for oral problems

Figure 3 shows the way that survey participants dealt with the oral issues. We can see that nearly half of the choices were simply tolerating these problems without any treatment. Taking medicine accounted for 29.1% of the choices. The other survey questionnaire choices were seeking emergency dental services (10.49%), online consultation with a dental institution (10.09%) and searching for folk prescriptions (6.75%). A total of 39.38% of the participants felt worried because of the unavailability of timely dental services, and 55.43% of the study population believed that they would take more care of their oral health after the epidemic.

### Impact of Covid-19 on future dental treatment

**Fig. 4:**
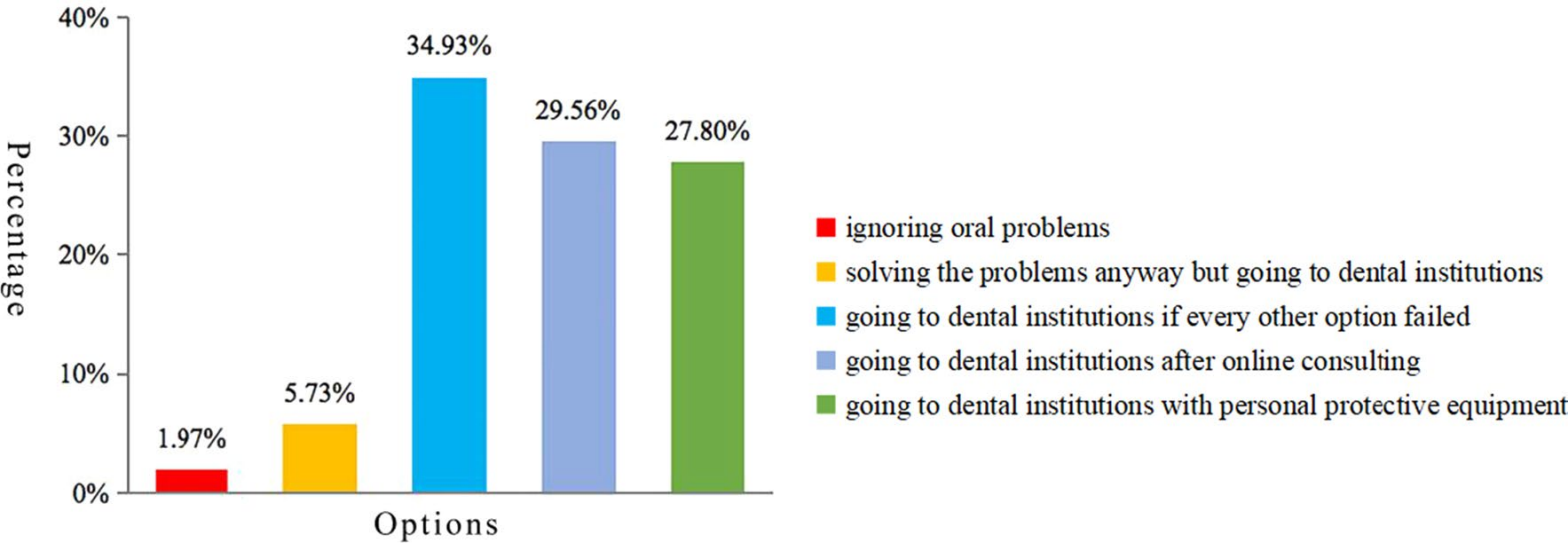
Different options for post-epidemic dental treatment

Two questions were raised about knowledge and attitudes towards possible nosocomial infection from dental treatment. Table 3 shows the age-specific participant responses from Wuhan and other places in China. A total of 73.33% of the participants knew that dental procedures could cause nosocomial infections. The percentage was significantly higher in Wuhan (76.38%) than in other places (70.84%). Regarding the safety of future dental treatment, 63.90% of the study population was concerned. Likewise, more participants in Wuhan showed insecurity than in other places in all three age groups. When asked about the option to deal with future oral problems, the majority of the study population chose not to go to professional dental institutions until they had to (34.93%), after online consultation (29.56%) and with proper personal protective equipment (27.80%) while the rest of the participants (7.70%) insisted on not going to hospitals for help (Fig. 4).

**Table 3:** Knowledge and attitudes about dental treatment and transmission of pathogens

	Population	Did you know that dental procedures could transmit pathogens? (%)		Are you concerned about the safety of dental treatment? after the epidemic? (%)		
		Yes	No	Concerned	Not concerned	Never thought about it
Wuhan	1507	76.38*	23.62	70.07*	12.01	17.92
Other places	1845	70.84	29.16	58.86	19.08	22.06
Total	3354	73.33	26.67	63.90	15.90	20.20

*p < 0.05 compared with other places

## Discussion

This study aimed to evaluate the impact of COVID-19 on the oral health of adults with a nationwide online cross-sectional questionnaire survey. The COVID-19 epidemic is a global health crisis, unlike any in modern history [12], which severely affects physical and mental health [13, 14]. Although the epidemic seems to be temporarily contained in China, it is still spreading worldwide. Experts from WHO warned of the second outbreak of this epidemic, and this virus may never entirely go away [15]. In this manner, the present study not only contributes to a comprehensive understanding of the impact of COVID-19 on oral health in China but may also provide additional advice for oral health maintenance during the epidemic for residents in other regions as well as for post-epidemic dental treatment.

The results of our survey show that the epidemic did affect psychological status and change living habits. We found that the participants in Wuhan tended to be more affected than those in other places in China. This finding is reasonable because Wuhan was the earliest epicentre of this epidemic. People in Wuhan may have suffered more from stress and other negative emotions. Previous studies have evaluated the mutual impact of psychological emotions and oral health [16–18]. In our study, the significantly higher prevalence of oral problems among participants in Wuhan may also be proof of this mutual correlation.

Toothbrushing frequency did not significantly differ between the participants of different regions and age groups. Notably, 73.09% of the total participants brushed their teeth twice or more a day. In 2015, the fourth national oral health survey was conducted in China. According to the results of that survey, 47.8% of the 35–44-year-old group and 30.6% of the 55–64-year-old group brushed their teeth twice or more a day [19, 20]. Although the age groups differed, the result could be a reference to indicate that the majority of the participants in the present survey had good oral hygiene behaviour. This result may be related to the fact that people might pay more attention to oral healthcare because of the inability to seek timely dental treatment. Educational background and socioeconomic status may also be factors influencing oral health behaviours. These factors require a more detailed questionnaire design and analysis in future research.

Compared with other common chronic diseases, the highest proportion of participants with a history of oral diseases felt that the original disease had worsened during the epidemic. This outcome illustrates the specificity of dental treatment in that the majority of oral problems can only be solved by professional dental procedures. Most dental institutions cannot provide regular dental services amidst the epidemic, leading to the worsening of existing oral problems.

Gingival bleeding, bad breath and oral ulcers were the three most common oral problems the study population encountered amid the epidemic. All three oral problems are associated with oral hygiene [21–24], and psychological status has been proven to be an important risk factor for oral ulcers [25]. To study the relationship between oral hygiene habits and oral disease further, we found that the prevalence of common oral diseases was the lowest among participants who brushed their teeth twice or more a day. All these findings suggest the importance of good oral hygiene behaviours and mental states in reducing the occurrence of oral diseases.

Inadequate dental services and reluctance to go outside made tolerance the most popular choice for the participants facing oral problems. People chose to stay home as much as possible because of the official recommendation and fear of the epidemic. This finding is in accordance with the results of a previous study showing significantly reduced dental emergency patients in Beijing in February 2020 [26].

Forty percent of the participants worried about inadequate dental services, which may have increased uneasiness and anxiety during the epidemic. Thus, we can see that more than half of the participants tended to pay more attention to oral healthcare after the epidemic. This result suggests that COVID-19 may raise public awareness about oral health while posing a great threat to people’s lives and health.

More than 70% of the participants understood that oral treatment may lead to the spread of infections. This response reflected people’s concerns about the transmission of this serious virus, and most people had correct knowledge. We found that more than 70% of the participants expressed concerns about the safety of dental treatment, and more participants in Wuhan were concerned compared with other places, which is also in accordance with the aforementioned finding that participants in Wuhan were more affected by the epidemic.

Despite this concern, 92.30% of the participants surveyed said they would go to dental institutions when facing dental problems after the epidemic. This response is an important message that reminds us of the importance of avoiding nosocomial infections in the post-epidemic period. Several studies have provided guidelines for the provision of dental care both during and after the epidemic, including the screening and assessment of patients, prevention of infection, hand hygiene, and personal protective equipment [27–29]. These guidelines remain to be further improved and evaluated. The most important thing is to abide strictly by the relevant regulations and guidelines during regular dental procedures; thus, both the patients and the dentists will be protected and safe.

## Conclusions

Based on the findings of our present survey, we could conclude that individuals in Wuhan tended to be more seriously affected and suffered more oral problems amidst the epidemic compared with people from other places in China. Keeping good oral health behaviours and mental status play an important role in preventing dental problems. It is crucial to establish and to follow the standard guidelines for the provision of dental care both during and after the epidemic.

Our research is a preliminary study of the impact of COVID-19 on oral health. This study is limited by the total sample size, and the population sampled may not be representative. More well-designed studies would contribute to a continuing understanding of this important issue.

## Supplementary Information

**Additional file 1:** The questionnaire for this survey.

## Data Availability

The datasets used and analysed during the current study are available from the corresponding author upon reasonable request.

## Abbreviations

PHEIC: Public Health Emergency of International Concern
SARS-CoV-2: Severe acute respiratory syndrome coronavirus-2
COVID-19: Coronavirus disease 2019
SAS: Self-Rating Anxiety Scale
