# Supplementary material for: Impact of COVID-19 on the oral health of adults in Wuhan and China: results of a nationwide online cross-sectional questionnaire survey
Source: BMC Oral Health. 2021 Mar 26;21:162. doi: 10.1186/s12903-021-01533-z (PMC7994179; doi:10.1186/s12903-021-01533-z)
Supplement: Supplementary file 1 — Additional file 1: The questionnaire for this survey. [file 12903_2021_1533_MOESM1_ESM.docx]

**Questionnaire about the impact of COVID-19 on oral health of adults in Wuhan and China**

1. Your gender

A male

B female

2. Your age

A 18-30 years old

B 31-40 years old

C 41-50 years old

D 51-60 years old

E 61-70 years old

F over 70 years old

3. What is your city during the epidemic period?

4. Do you feel nervous and anxious during the epidemic?

A never

B sometimes

C often

D always

5. Do you feel upset and frightened during the epidemic?

A never

B sometimes

C often

D always

6. Do you feel difficult to fall asleep or sleep poorly during the epidemic?

A never

B sometimes

C often

D always

8.Did your daily work and rest time change during the epidemic than before?

A yes

B no

9. How did your frequency of meals change during the epidemic compared with before?

A increases

B decreases

C unchanged

10. How did your consumption of cigarettes change during the epidemic compared with before？

A increased

B decreased

C unchanged

D I do not smoke

11. How did your consumption of alcohol change during the epidemic compared with before？

A increased

B decreased

C unchanged

D I do not drink alcohol

12. What is the frequency of your toothbrushing during the epidemic?

A twice or more a day

B once a day

C less than once a day

13.How did your frequency of toothbrushing change during the epidemic compared with before?

A increases

b decreases

C unchanged

14.Are you more concerned about hygiene and oral health during the epidemic than before?

A more concerned

B. unchanged

C less concerned

D never concerned

15. Did you have the following diseases or related symptoms before the epidemic？ (You can choose one answer or more)

A diabetes

B cardio cerebrovascular disease

C chronic respiratory diseases

D oral diseases

E none of the above

F I don't know

16.What are the changes of your original disease and related symptoms during the epidemic？

A. severe

B. remission

C. no change

17. Did you have the following diseases or related symptoms during the epidemic？ (You can choose one answer or more)

A diabetes

B cardiovascular and cerebrovascular disease

C chronic respiratory diseases

D oral diseases

E none of the above

I don't know

18. DID you have the following oral problems during the epidemic period? (You can choose one answer or more)

A gingival bleeding

B swelling

C toothache

D Inflammation of wisdom teeth

E. bad breath

F oral ulcer

G TMJ disorders (pain, snapping, limited mouth opening)

H other oral problems

I I did not have any oral problems

19.When you encountered the above oral problems, what did you do?

A tolerating and ignoring

B. searching for prescription

C online consultation with dental institutions

D seeking emergency dental service

E taking medicine

F other options

20. Do you know dental procedures could transmit pathogens?

A yes

B no

21. Do you concern about the safety of dental treatment after the epidemic?

A Concern

B Do not concern

C Never thought about it

22.Will you pay more attention to oral health care and oral disease prevention after the epidemic?

A Will pay more attention

B No change,

C Will pay less attention

D Never thought about it

23.When you have oral problems after the epidemic, what will you do?

A ignoring the oral problems

B solving the problems anyway but going to dental institutions

C going to dental institutions if everyway else fails

D going to dental institutions after online consulting with them

E going to dental institutions with personal protective equipments
